# Down-Regulation of *PpBGAL10* and *PpBGAL16* Delays Fruit Softening in Peach by Reducing Polygalacturonase and Pectin Methylesterase Activity

**DOI:** 10.3389/fpls.2018.01015

**Published:** 2018-07-11

**Authors:** Hangkong Liu, Ming Qian, Chunhui Song, Jinjin Li, Caiping Zhao, Guofang Li, Anzhu Wang, Mingyu Han

**Affiliations:** ^1^College of Horticulture, Northwest A&F University, Yangling, China; ^2^Centre of Pear Engineering Technology Research, State Key Laboratory of Crop Genetics and Germplasm Enhancement, Nanjing Agricultural University, Nanjing, China

**Keywords:** peach, β-galactosidases, virus-induced gene silencing (VIGS), softening, polygalacturonase, pectin methylesterase

## Abstract

β-galactosidases are cell wall hydrolases that play an important role in fruit softening. However, *PpBGAL*s mechanism impacting on ethylene-dependent peach fruit softening was still unclear. In this study, we found that *PpBGAL4, -6, -8, -10, -16*, and -*17* may be required for ethylene-dependent peach softening and *PpBGAL10*, -*16* may make a main contribution to it among 17 *PpBGALs*. Utilization of virus-induced gene silencing (VIGS) showed that fruits were firmer than those of the control at 4 and 6 days after harvest (DAH) when *PpBGAL10* and *PpBGAL16* expression was down-regulated. Suppression of *PpBGAL10* and *PpBGAL16* expression also reduced *PpPG21* and *PpPME3* transcription, and polygalacturonase (PG) and pectinmethylesterases (PME) activity. Overall, total cell wall material and protopectin slowly declined, water-soluble pectin slowly increased, and cellulose and hemicellulose was altered significantly at 4 DAH, relative to control fruit. In addition, *PpACO1* expression and ethylene production were also suppressed at 4 DAH because of inhibiting *PpBGAL10* and *PpBGAL16* expression. These results suggested that down-regulation of *PpBGAL10* and *PpBGAL16* expression delays peach fruit softening by decreasing PG and PME activity, which inhibits cell wall degradation and ethylene production.

## Introduction

Peach (*Prunus persica* [L.] Batsch) is a typical climacteric fruit that readily softens after harvest (Yoshioka et al., 2010). The short shelf-life of peaches decreases their market value and represents a major factor limiting the expansion of the fresh market peach industry. Fruit ripening and softening is a complex and coordinated process which is usually accompanied by changes in firmness, color, and flavor (Osorio et al., 2013). Many studies have reported that the process of fruit softening is related to cell wall modifications involving depolymerization of pectins and matrix glycans, solubilization of pectin polymers, and the loss of neutral sugars from pectin side chains (Ruiz May and Rose, 2013; Tucker, 2014; Paniagua et al., 2016). Enzymes related to cell wall modifications that potentially play a role in fruit softening include polygalacturonase (PG; EC3.2.1.15), pectin methylesterases (PME; EC3.1.1.11), β-galactosidase (β-gal; EC3.2.1.23), cellulase (EC3.2.1.4), and xyloglucan endotransglycosylase (EC2.4.1.207) (Hinton and Pressey, 1974; Lazan et al., 2004; Belleau-Deytieux et al., 2009; Qian et al., 2016). β-Gal increases cell wall porosity by depolymerizing galactose side chains of xyloglucan, rhamnogalacturonan I, and hemicelluloses, which allows binding of PG, PME, or other cell wall hydrolases to pectin; consequently accelerating fruit softening (Brummell and Harpster, 2001; Gerardi et al., 2012; Pose et al., 2013).

In plants, β-gals belong to the glycoside hydrolase 35 family. β-*gal* genes have been identified in *Arabidopsis thaliana* (Ahn et al., 2007), tomato (Smith and Gross, 2000), Japanese pear (Tateishi et al., 2005), *Brassica campestris* (Liu et al., 2013), and peach (Guo et al., 2018). More specifically, the transcript abundance of 17 Arabidopsis *β-gal* genes was measured by q-PCR in five tissues: leaves, roots, flowers, green seedlings, and etiolated seedlings (Ahn et al., 2007). In tomato, seven *TBGs* were found to be expressed in fruits, four in leaves and flowers, five in roots, and six in stems (Smith and Gross, 2000). Similar observations have been reported in Japanese pear (Tateishi et al., 2005) and *B. campestris* (Liu et al., 2013). These studies have described the tissue-specific expression of plant β-*gal*s and their extensive functional divergence. Previous studies have also indicated that β-*gals* contribute to a variety of biological processes, including fruit softening (Pressey, 1983; Carey et al., 1995; Smith et al., 2002), flower senescence (Raghothama et al., 1991), fruit abscission (Wu and Burns, 2004), cell wall loosening (Dopico et al., 1989), galactolipid turnover (?), and xyloglucan mobilization (de Alcântara et al., 1999).

Several studies have specifically focused on the role of β-*gals* during fruit softening. *Fa*β*gal1* in strawberry (*Fragaria* × *ananassa*) displayed a softening-associated expression pattern with peak transcript levels in red fruit (Trainotti et al., 2001). In another study, inhibition of *Fa*β*Gal4*, which is expressed mainly in receptacles during strawberry fruit ripening, resulted in silencing of *Fa*β*Gal1*, which resulted in an increase in the amount of covalently bound pectin and fruit that was 30% firmer than control fruit (Paniagua et al., 2016). Smith et al. (2002) found that four of six antisense lines with down-regulated *TBG4* produced significantly firmer tomato fruit than control fruit. One line had lower *TBG4* mRNA levels and exo-β-gal activity and higher galactosyl content, suggesting that *TBG4* is involved in cell wall modifications associated with fruit softening (Smith et al., 2002). Similar results have been reported for *pPGBII* in papaya (Othman et al., 2011) and *MA-Gal* in banana (Zhuang et al., 2006).

As a plant hormone, ethylene plays a significant role in fruit softening (Hayama et al., 2006; Khan and Singh, 2009; Harb et al., 2012; Bu et al., 2013; Tatsuki et al., 2013). Many studies about β-*gal* genes mainly focus on the ethylene-dependent fruit softening. *PpGAL1* and *PpGAL4* may play a crucial role in ‘LaFrance’ pear softening, and their expression was up-regulated by exogenous ethylene or down-regulated by 1-MCP (1-Methylcyclopropene) (Mwaniki et al., 2005). In antisense-ACO melon, ethylene was found to be suppressed to less than 0.5% of the level in control fruit, with a concomitant decrease in β-*gal* gene expression (Nishiyama et al., 2007). Ban et al. (2016) also found that *DkGAL1* in persimmon participating in fruit softening could be regulated by ethylene. In addition, investigations of β-*Gal* in apple, *TBG4* in wild-type tomato, two ripening-impaired tomato mutants (rin and Nr), and *AV-GAL1* in avocado, have all strongly suggest that a regulative mechanism exists between ethylene and β-*gals* during ethylene-dependent fruit softening (Moctezuma et al., 2003b; Tateishi et al., 2007; Wei et al., 2012). However, the regulative mechanism between ethylene and β-*gal* genes during ethylene-dependent fruit softening was still unclear.

Rapid fruit softening in peach is a significant problem that affects fresh-market production. The molecular regulation of softening in peach, however, is still unclear. Although the importance of β-*gals* in fruit ripening and softening has been documented in many previous studies, the study about *PpBGALs* in peach is limited in the report which 17 *PpBGALs* (*PpBGAL1-17*) were only be identified by bioinformatics methods and displayed divergent expression during softening of four different peach cultivars (Guo et al., 2018). However, little is known about the roles of *PpBGALs* in ethylene-dependent peach softening. This includes characterizing which ones exhibit softening-associated expression patterns and how they may be involved in the regulation of fruit softening in peach. In the present study, we profiled the expression of 17 *PpBGALs* coming from the study of Guo et al. (2018) in response to propylene and 1-MCP treatments during peach fruit softening. *PpBGALs* exhibiting consistent softening-associated expression patterns were identified, and the function of *PpBGAL10* and *PpBGAL16* in peach fruit softening was explored using virus-induced gene silencing (VIGS). The overall objective was to develop a better understanding of the molecular mechanisms by which *PpBGALs* regulate ethylene-dependent peach fruit softening.

## Materials and Methods

### Plant Material and Treatments

‘Qian jian bai’ (QJB) peach trees, grown at the Experimental Station of the College of Horticulture, Northwest Agriculture and Forestry University, Yangling, Shaanxi, China were used in this study. Fruits were harvested at commercial maturity (exhibiting partially red, light-green skin and slightly firm flesh; Qian et al., 2016) and transported to the laboratory. Undamaged fruits were selected and divided randomly into three groups, each containing 150 fruits. Each group was then sub-divided into three additional groups. Fruits in the first and second group were placed in hermetic containers and treated for 24 h with 500 μL L^−1^ propylene or 5 μL L^−1^ 1-MCP, respectively. Propylene treatment can eliminate interference of exogenous ethylene when endogenous ethylene production of peach fruit is measured by gas chromatography (Trace GC Ultra, Thermo Fisher, New York, NY, United States). The third group of fruit was sealed in a hermetic container with air for 24 h as control. Following treatment, fruits from each of the groups were stored at 25°C and 75% relative humidity. Fruit samples were taken every other day until they were fully softened and at each sampling the fruit were frozen rapidly in liquid nitrogen and stored at −80°C until further analysis.

### Determination of Fruit Firmness, Ethylene Production, and Enzyme Activity

Fruit firmness of five randomly selected fruits from each sub-group receiving each treatment was measured using a GY-4 firmness meter (Top Instrument Co., Hangzhou, China) equipped with a 7.9-mm probe. The skin of the peel was removed from a section of the fruit surface and a probe was inserted and the pressure it required to penetrate the flesh of the fruit was recorded. Ethylene production was analyzed as described by Liguori et al. (2004). Briefly, nine fruits from each sub-group in each treatment were sealed in a jar for 60 min, and a 1-mL air sample was analyzed by gas chromatography (Trace GC Ultra, Thermo Fisher, New York, NY, United States). The enzyme activity of β-gal, PG, and PME in 1 kg fresh weight (FW) peach flesh was determined as reported by Gross (1982), Lazan et al. (1989), and Hagerman and Austin (1986), respectively. One unit (U) of β-gal and PG enzyme activity was defined as the amount of hydrolyzed enzyme producing 1 mol p-nitrophenol and galacturonic acid per minute, respectively. One unit of PME enzyme activity was defined as the amount of enzyme producing 1 μmol CH_3_O^-^ by de-methylesterification per minute. Separation and measurement of cell wall materials (dry mass) was performed as described by Santiago-Domenech et al. (2008). Each experiment was carried out in three replicates.

### Cloning of *PpBGAL10* and *PpBGAL16* and Virus Induced Silencing (VIGS)

*PpBGAL10* and *PpBGAL16* came from previous report (Guo et al., 2018), gene-specific primers used to clone their coding sequences were designed using Primer Premier 6.0 (Supplementary Table S1). Restriction enzyme cutting sites and protective bases were added to the forward and reverse primers. Each 50-μL PCR amplification mixture contained 1 μL high-fidelity DNA polymerase (Vazyme, Nanjing, China), 10 μL buffer, 1 μL dNTPs, 5 μL cDNA template, 3 μL each of the forward and reverse primers, and 27 μL sterilized double-distilled H_2_O. Amplifications were performed on a GeneAmp PCR System 9700 (ABI, Waltham, MA, United States) using the following cycling conditions: 2 min at 95°C, followed by 40 cycles of 10 s at 95°C, 30 s at the selected annealing temperature, and 15 s at 72°C, with a final extension of 10 min at 72°C. The PCR products were subjected to electrophoresis on 1% agarose gels and then inserted in a pMD18-T vector (Takara, Dalian, China) for sequencing. After verifying the coding sequence, the target gene was cloned into a pTRV2 vector. The two recombinant plasmids (pTRV2-*PpBGAL10* and pTRV2-*PpBGAL16*), as well as a control (a pTRV2 empty plasmid) were separately introduced into *Agrobacterium tumefaciens* GV3101 using a freeze-thaw method (Fire et al., 1998). Individual colonies were subsequently incubated overnight at 28°C in 1 mL LB medium containing 50 mg mL^−1^ kanamycin, 50 mg mL^−1^ gentamicin, 50 mg mL^−1^ rifampicin, 20 mM acetosyringone, and 10 mM MES. An aliquot of each culture was then inoculated into 100 mL of the same antibiotic LB medium and incubated to an *A*_600_ of 1.0–2.0 at 28°C. *Agrobacterium* infection was performed according to the method of Jia et al. (2011). Cells were collected by centrifugation at 5000 × *g* and 25°C for 5 min and then resuspended in an equal volume of infiltration buffer containing 10 mM MgCl_2_, 200 μM acetosyringone, and 10 mM MES (pH 5.6) and incubated at 25°C for 3 h. Finally, 1 mL of a 1:1 (v/v) mixture of induced *Agrobacterium* harboring pTRV2, and *Agrobacterium* with either pTRV2-*PpBGAL10* or pTRV2-*PpBGAL16*, was infiltrated into fruit using a 1-mL syringe. Fruit were infiltrated at nightfall when the bacterial culture was at the end of the second exponential growth phase. Infiltrated peach fruit of three constructs was picked at 1 week after infiltration and stored at 25°C and 75% relative humidity, respectively (Li et al., 2017). Each construct contains 150 fruits and then divided equally into three sub-groups. Fruit samples of each sub-groups were taken every other day until control fruit fully softening, and stored at −80°C after freezing quickly in liquid nitrogen. The ethylene production of infiltrated fruit and in other experiments (including fruit firmness, gene expression, enzyme activity, and cell wall components) at the infected position were performed using the above-mentioned methods.

### RNA Extraction and Reverse Transcription

Total RNA was extracted as described by Lester et al. (1994). RNA quality and integrity were determined using 1% agarose gel electrophoresis and ultraviolet spectrophotometry (Thermo NanoDrop 2000, Wilmington, DE, United States). Reverse transcription was conducted using a Prime Script RT Reagent Kit with gDNA Eraser (TaKaRa, Dalian, China).

### Reverse Transcription-Quantitative PCR (RT-qPCR)

Specific primers for 17 *PpBGALs* coming from previous report (Guo et al., 2018), *PpPG21*, *PpPME3*, *PpACS2*, and *PpACO1* were designed using Primer Premier 6.0 (Qian et al., 2016; Li et al., 2017) (Supplementary Table S1). RT-qPCR analyses were conducted using an iQ5 real-time PCR system (Bio-Rad, Plano, TX, United States). A 10-μL reaction volume was used for each sample comprising 1 μL cDNA, 1 μL of each primer, 2 μL ddH_2_O, and 5 μL of 2 × SYBR Premix Ex *Taq* II (TaKaRa, Dalian, China). The PCR protocol specified in the SYBR Premix Ex *Taq* kit manual was as follows: 1 min at 95°C, followed by 40 cycles of 15 s at 95°C, 20 s at the selected annealing temperature, and 20 s at 72°C, followed by 10 s at 95°C, and finally 39 cycles to construct a melting curve. The peach 18S ribosomal RNA (rRNA) gene was used as a reference gene and for normalization of the data. Relative expression levels for each of the analyzed genes were calculated as described by Livak and Schmittgen (2001). Each sample was composed of three biological replicates.

### Statistical Analysis

Microsoft Excel 2010 and IBM SPSS Statistics 22 were used for data processing and to determine significant statistical differences between sample representing different time points and treatments using *post hoc* Tukey’s test of One-way ANOVA (*p* < 0.05) for differences. Figures were generated and combined using Sigma Plot 10.0.

## Results

### Fruit Firmness, Ethylene Production, and β-Gal Activity During Peach Fruit Softening

**FIGURE 1:**
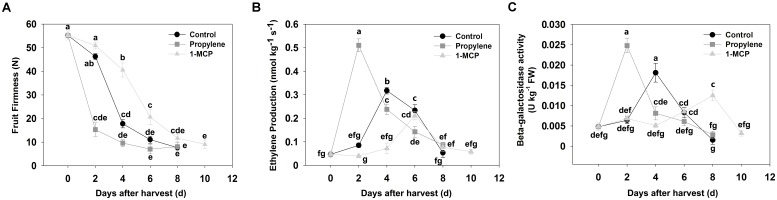
Fruit firmness, ethylene production, and β-galactosidase activity in control, propylene-treated, and 1-MCP-treated ‘Qian Jian Bai’ peach fruit during storage. **(A)** Fruit firmness; **(B)** ethylene production; **(C)** β-gal activity. Each experiment was repeated three times. Data represent the mean ± SE (*n* = 3). Significant differences (*p* < 0.05) between means are indicated by different letters.

Fruit firmness in QJB control fruit decreased slowly over the first 2 days after harvest (DAH), declined rapidly from 2 to 4 DAH, and then decreased slowly (**Figure 1A**). Ethylene production increased slowly during the first 2 DAH, increased significantly from 2 to 4 DAH, and then rapidly decreased in subsequent DAH (**Figure 1B**). Changes in β-gal activity exhibited a similar trend after harvest to ethylene production, with maximum β-gal activity observed at 4 DAH (**Figure 1C**).

### Identification of *PpBGALs* With a Ripening-Associated Pattern of Expression

**FIGURE 2:**
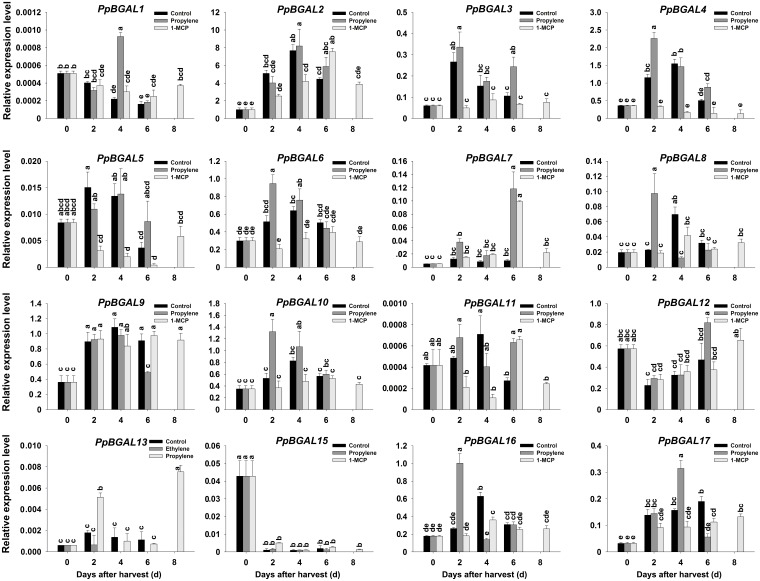
Transcript levels of 17 *PpBGALs* in control, ethylene-treated, and 1-MCP-treated ‘Qian Jian Bai’ peach fruit during storage. The peach 18S rRNA gene was used as a reference. The experiment was repeated three times. Data represent the mean ± SE (*n* = 3). Significant differences (*p* < 0.05) between means are indicated by different letters.

RT-qPCR was used to analyze the expression profiles of 17 *PpBGALs* during QJB fruit softening to provide information on the potential role of *PpBGALs*. Among the *PpBGALs* examined, *PpBGAL2, -4, -6, -8, -9, -10, -16*, and -*17* were up-regulated and exhibited their maximum expression level at 4 DAH, with the exception of *PpBGAL17* which exhibited peak transcript levels at 6 DAH (**Figure 2**). However, *PpBGAL15* exhibited a tendency to be down-regulated, and *PpBGAL12* firstly decreased in the peach fruit and then increased (**Figure 2**). *PpBGAL3* and -*7* were up-regulated during the first 2 DAH and then down-regulated; *PpBGAL1, -5, -11*, and -*13* were barely detected while *PpBGAL14* expression was not detected during QJB softening (**Figure 2**).

### Propylene and 1-MCP Treatments Alter β-Gal Expressions

The QJB peach fruits were treated with propylene and 1-MCP to determine the potential role of *PpBGAL* family members during ethylene-dependent fruit softening. Fruit firmness decreased markedly at 2 DAH in response to the propylene treatment (**Figure 1A**). Correspondingly, ethylene production and PpBGAL activity increased rapidly during the first 2 DAH (**Figures 1B,C**). *PpBGAL4, -6, -8*, -*10*, and -*16* expression was significantly up-regulated and peaked at 2 DAH in propylene-treated fruit; similarly, *PpBGAL1*, -*17* and *PpBGAL7* expression also increased significantly but peaked at 4 or 6 DAH, respectively (**Figure 2**). Transcript levels of *PpBGAL2, -3, -5, -9, -11, -12, -13*, and -*15* were not significantly affected by the propylene treatment (**Figure 2**).

Fruits treated with 1-MCP softened more slowly relative to non-treated control fruit (**Figure 1A**). Ethylene production was also lower relative to the control fruit at 4 DAH, with peak ethylene levels exhibited at 6 DAH (**Figure 1B**); β-gal activity was significantly inhibited at same time, with maximum activity exhibited at 8 DAH (**Figure 1C**); Expression of *PpBGAL 2, -3, -4, -5, -6, -8, -10, -11, -16*, and -*17* was inhibited, while transcript levels of *PpBGAL1, -7, -9, -12*, -*13*, and -*15* were barely affected (**Figure 2**).

### VIGS of *PpBGAL10* and *PpBGAL16*

**FIGURE 3:**
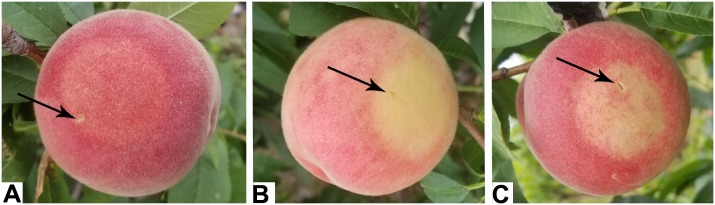
Phenotypes of infiltrated fruits. **(A)** TRV2 (control); **(B)** TRV2-*PpBGAL10* (RNAi-10); **(C)** TRV2-*PpBGAL16* (RNAi-16). Black arrows indicate the injection site.

**FIGURE 4:**
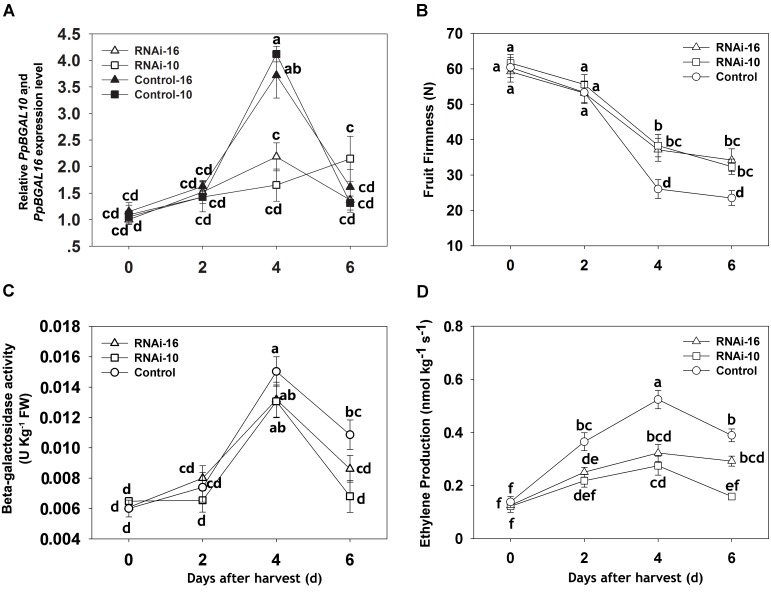
Changes in the expression level of *PpBGAL10* and *PpBGAL16*, fruit firmness, β-gal activity, and ethylene production during storage of control (TRV2), RNAi-10 (TRV2-*PpBGAL10*), and RNAi-16 (TRV2-*PpBGAL16*) fruit. **(A)** Relative transcript abundance of *PpBGAL10* and *PpBGAL16*, RNAi-10, RNAi-16 and Control-10, Control-16 represents expression of *PpBGAL10* and *PpBGAL16* in RNAi and Control fruit, respectively, **(B)** fruit firmness, **(C)** β-gal activity, and **(D)** ethylene production RT-qPCR expression levels were normalized using the cycle threshold value of the peach 18S rRNA gene. Data represent the mean ± SE (*n* = 3). Significant differences (*p* < 0.05) between means are indicated by different letters.

Virus-induced gene silencing technology was used to suppress the expression of *PpBGAL10* and *PpBGAL16* (RNAi-10 and RNAi-16, respectively) in fruit tissues to confirm the roles of these genes in peach fruit softening. The infiltrated surfaces of control fruits developed a typical red flush, whereas little or no red color was evident at the areas of fruit infiltrated with RNAi-10 and RNAi-16 (**Figure 3**). Expression of *PpBGAL10* and *PpBGAL16* was significantly decreased at 4 DAH in RNAi fruit (**Figure 4A**). The fruits infiltrated with the two RNAi constructs softened more slowly, as measured by changes in fruit firmness, during the period of 2–6 DAH than control fruit infiltrated with an empty vector construct (**Figure 4B**). Total β-gal activity, however, was not significantly different between the fruit infiltrated with the RNAi constructs and the control fruit from 0 to 4 DAH (**Figure 4C**).

**FIGURE 5:**
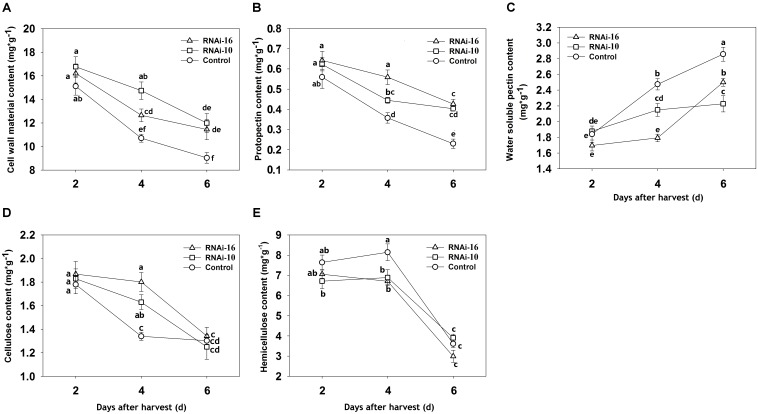
Changes in the level of cell wall components in control (TRV2), RNAi-10 (TRV2-*PpBGAL10*), and RNAi-16 (TRV2-*PpBGAL16*) fruit. **(A)** Cell wall material (dry mass), **(B)** protopectin, **(C)** water-soluble pectin, **(D)** cellulose, and **(E)** hemicelluloses in RNAi and control fruit during storage. Each experiment was repeated three times. Bars represent the mean ± SE (*n* = 3). Significant differences (*p* < 0.05) between means are indicated by different letters.

The amounts of various cell wall components (cell wall material, protopectin, water-soluble pectin, hemicellulose, and cellulose) were different in the RNAi constructs fruit than in the control fruit (**Figure 5**). In RNAi constructs fruit, the amount of cell wall material (dry mass), protopectin, and cellulose were greater at 4 DAH (**Figures 5A,B,D**). Although water-soluble pectin content increased from 2 to 6 DAH in both the control and RNAi, the increase was greater in the control (**Figure 5C**). Interestingly, hemicellulose content was higher in the control fruit than in RNAi fruit from 2 to 4 DAH and then decreased sharply in all three groups (**Figure 5E**).

**FIGURE 6:**
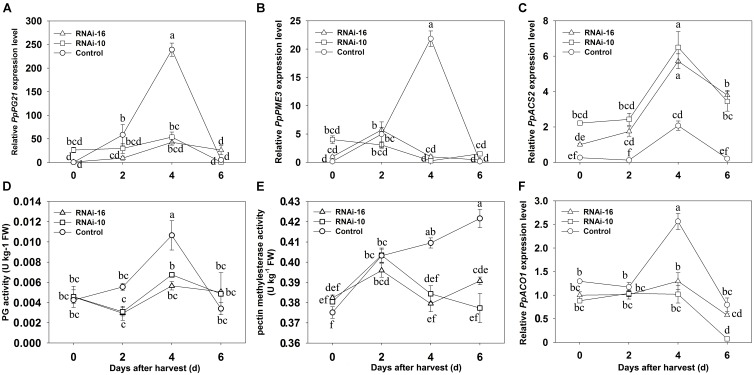
Changes in softening-related gene expression and cell wall hydrolase activity in control, RNAi-10, and RNAi-16 fruit during storage. **(A)** Relative expression of *PpPG21*; **(B)** relative expression of *PpPME3*; **(C)** relative expression of *PpACS2*; **(D)** polygalacturonase activity; **(E)** pectin methylesterase activity; and **(F)** relative expression of *PpACO1*. Each experiment was repeated three times. Data represent the mean ± SE (*n* = 3). Significant differences (*p* < 0.05) between means are indicated by different letters.

Transcript levels of softening-related genes (*PpPG21* and *PpPME3*) and the enzyme activity of cell wall hydrolases (PG and PME) were measured in control and RNAi fruits from 0 to 6 DAH (**Figure 6**). Expression of *PpPG21* and *PME3* reached their maximum at 4 DAH in control fruit and was significantly higher than in RNAi fruit, but no significant differences were observed in the expression of these genes between RNAi-10 and RNAi-16 fruit from 0 to 6 DAH (**Figures 6A,B**). PG maximum activity was higher in the control fruit though peaked at 4 DAH in both control and RNAi fruit (**Figure 6D**). PME activity, which peaked at 2 DAH in RNAi fruit, increased slowly in control fruit from 2 to 4 DAH and was higher at 4 and 6 DAH than in the RNAi fruit (**Figure 6E**).

### Down-Regulation of *PpBGAL10* and *PpBGAL16* Affects Ethylene Production and Ethylene-Related Gene Expression

The contribution of ethylene to the softening of respiratory climacteric fruit is well known. In the present study, ethylene production and transcript levels of ethylene-related genes (*ACO1* and *ACS2*) were analyzed in RNAi and control fruits from 0 to 6 DAH. As illustrated in **Figure 4D**, ethylene production at 4 DAH was significantly lower in RNAi fruit than in control fruit, however, *PpACS2* transcript levels were higher in RNAi fruit from 0 to 6 DAH. In addition, *PpACS2* expression was similar in both types of RNAi fruit (**Figure 6C**). Interestingly, *ACO1* expression level was significantly higher at 4 DAH than in either of the two different RNAi fruit that exhibited similar levels of expression to each other (**Figure 6F**).

## Discussion

### Possible Role of *PpBGAL* Family Members in Fruit Softening

Several studies have focused on the possible role of β-gals in fruit ripening and softening (Smith et al., 2002; Lazan et al., 2004; Yoshioka et al., 2011; Paniagua et al., 2016). Guo et al. (2018) reported three *PpBGAL* genes (*PpBGAL2, -8* and -*16*) in ‘Hu Jing Mi Lu’ and five *PpBGAL* genes (*PpBGAL1, -2, -9, -12*, and -*16*) in ‘Xia Hui 8’ peach fruit were up-regulated during storage. However, results of the present study indicate that *PpBGAL2, -4, -6, -8, -9, -10*, -*16*, and -*17* may participate in QJB fruit softening due to exhibit softening-associated patterns of expression, with transcript levels being up-regulated during the process of fruit softening in QJB peach fruit (**Figure 2**). Therefore, it appears that several *PpBGALs* could involve in peach fruit softening while their expression can vary between different peach cultivars.

Our results also indicate that, *PpBGAL4, -6, -8, -10*, -*16*, and -*17* can be induced by endogenous ethylene (**Figure 2**), which has been reported to be increased by propylene treatment (Ban et al., 2016). Therefore, the six *PpBGALs* may play an important role in ethylene-dependent QJB fruit softening. In addition, *PpBGAL16* exhibited the same expression pattern in three different peach cultivars (‘Hu Jing Mi Lu,’ ‘Xia Hui 8,’ and QJB), and exhibits a low level of expression during the storage of ‘Yumyeoung’ and ‘XiaCui.’ Notably, both of these latter cultivars maintain fruit firmness for a longer period of time than the former three cultivars and barely synthesize any ethylene during storage (Guo et al., 2018). Therefore, it appears that *PpBGAL16* may play a pivotal role in ethylene-dependent peach fruit softening. *PpBGAL10* exhibited the pattern of expression as well as *PpBGAL16* in propylene-treated and control fruit (**Figure 2**). Meanwhile, it may be an ortholog of PpGAL3 has been reported to play a role in cell wall disassembly in ripening Japanese pear (Tateishi et al., 2005). Therefore, *PpBGAL10* may also play an important role in line with *PpBGAL16* during peach softening.

*PpBGAL2* and *PpBGAL9* may participate in QJB fruit softening in an ethylene-independent manner. The expression of *PpBGAL1, -5, -11*, -*13*, and -*14* were very low or undetectable in naturally softened QJB peach fruit, while *PpBGAL3, -7, -12*, and -*15* exhibited hardly showed soften-related expression patterns (**Figure 2**) and were only slightly induced by exogenous propylene; suggesting that they have negligible roles in ethylene-dependent peach fruit softening.

### Down-Regulation of *PpBGAL10* and *PpBGAL16* Delays Peach Fruit Softening

To further elucidate the functional role of *PpBGALs* in ethylene-dependent peach fruit softening, VIGS technology was utilized to suppress the expression of two principle *PpBGALs* (*PpBGAL10* and *PpBGAL16*) in fruit infiltrated with RNAi constructs. Results indicated that fruit softening was delayed in fruit infiltrated with both RNAi constructs (**Figure 4B**), however, β-gal activity was only slightly lower in the RNAi fruit (**Figure 4C**) when the expression of *PpBGAL10* and *PpBGAL16* was significantly down-regulated (**Figure 4A**). These results are consistent with studies in strawberry which found that the down-regulation of *Fa*β*Gal4* resulted in delayed fruit softening but no significant change in total β-Gal enzyme activity (Paniagua et al., 2016). Similar results have also been reported by Carey et al. (2001) and Smith et al. (2002) in tomato. We suggest that down-regulation of *PpBGAL10* and *PpBGAL16* may lead to reduced exo-β-galactanase activity, a change that would have a negligible effect on total β-Gal enzyme activity (Moctezuma et al., 2003a; Paniagua et al., 2016).

PG can depolymerize cell wall due to mediate homogalacturonan depolymerization requiring to be de-methylesterified by PME (Brummell and Harpster, 2001). Thus, PG and PME had been abundantly reported to contribute to fruit softening because of involving a role in cell wall metabolism (Micheli, 2001; Smith et al., 2002; Jayani et al., 2005; Payasi et al., 2009; Pose et al., 2013). β-gal increases cell wall porosity by depolymerizing the galactose side chains of xyloglucan, rhamnogalacturonan I, and hemicelluloses, which then allows the binding of PG, PME, or other cell wall hydrolases to pectin; thus accelerating fruit softening (Brummell and Harpster, 2001; Gerardi et al., 2012; Pose et al., 2013). Therefore, the activity of PG and PME in RNAi fruit might be affected by down-regulating expression of *PpBGAL10* and *PpBGAL16*. Our results indicated PpPG21 and PpPME3, two key genes encoding PG and PME, respectively, have significant lower expression in RNAi fruit than control fruit at 4 DAH, resulting in the reduction of PG and PME enzyme activity (**Figures 6A,B,D,E**). It is consistent with a viewpoint that β-galactosidase and ripening-related expansins may regulate other cell wall modify-related enzyme activities (Brummell and Harpster, 2001). These results suggest that the down-regulation expression of *PpBGAL10* and *PpBGAL16* delays peach fruit softening due to reduce PG and PME activity rather than β-gal activity.

### Down Regulation of *PpBGAL10* and *PpBGAL16* Impacts Cell Wall Components

Accompanied by rapid declining of fruit firmness, water-soluble pectin contents could dramatically increase during melting peach fruit softening (Murayama et al., 2009). A slower rate of increase in water-soluble pectin was observed in RNAi-10 and RNAi-16 fruit where PG and PME activity was inhibited (**Figure 5C**). This result is consistent with results reported in strawberry after the down-regulation of *FaPG1*, *PL*, and *Fa*β*Gal4* genes (Santiago-Domenech et al., 2008; Pose et al., 2013; Paniagua et al., 2016). In addition, A decrease of protopectin content was occurred during ‘Okubo’ peach softening (Li et al., 2009). Our results displayed its levels in RNAi-10 and RNAi-16 fruit were higher (**Figure 5B**). These data suggested that softening of RNAi-10 and RNAi-16 fruit was delayed because of suppressing pectin metabolism. Therefore, it was indicated that the amount of ionically and covalently bound pectin was potentially higher in RNAi-10 and RNAi-16 fruit than in control fruit. Yoshioka et al. (2011) found that bound pectin (ionically and covalently) content was the higher in non-softening peach fruit than in softening at different storage time. Santiago-Domenech et al. (2008) and Figueroa et al. (2010) have also confirmed the depolymerization of bound pectin may be due in part to the solubilization of pectin. Moreover, fruit softening in peach is associated with pectin solubilization and depolymerization (Yoshioka et al., 2011). Therefore, the present results suggest that the inhibition of *PpBGAL10* and *PpBGAL16* transcription helps to reduce bound pectin solubilization and depolymerization by suppressing PG and PME activity, thereby delaying peach softening. In addition, changes of cellulose and hemicellulose level indicate cellulase and hemicellulase may be also influenced in RNAi-10 and RNAi-16 fruit, suggesting delaying fruit softening is likely a cooperative process which many cell wall modified enzymes engage together, but this mechanism is unclear and still required to further study.

### Suppression of *PpBGAL10* and *PpBGAL16* Reduces Ethylene Production

Ethylene is a hormone that plays an essential role in fruit softening through its ability to regulate several cell wall hydrolysis-related genes (Hayama et al., 2006; Tatsuki et al., 2013). Therefore, a reduction in ethylene production may greatly delay fruit softening. Ethylene production was significantly reduced in the present study when the expression of *PpBGAL10* and *PpBGAL16* was down-regulated. We propose three hypotheses to explain the reduction in ethylene production. First, the level of cell wall galactose in RNAi-10 and RNAi-16 fruit was likely reduced due to the observed inhibition of PG and PME activity, delaying pectin solubilization and depolymerization. Galactose, as a signaling molecule, has been confirmed to stimulate ethylene production in tomato fruits and tobacco leaf disks (Kim et al., 1987; Philosoph-Hadas and Aharoni, 1987). Therefore, a reduction in galactose content may reduce ethylene production by suppressing the transcription of *PpACO1*. Second, specific wall fragments, oligogalacturonides (OGAs) which are short breakdown products of homogalacturonan consisting of 9–15 GalA residues, have been suggested to induce ethylene release during pectin solubilization and depolymerization (Simpson et al., 1998; Wolf et al., 2012), and PME-dependent demethylation-esterification of OGAs is essential to this process (Osorio et al., 2008). In the present study, the amount and demethylation of OGAs are thus probably reduced in RNAi-10 and RNAi-16 fruit where PME and PG activity is reduced. This scenario would also result in a reduction in ethylene production. A third hypothesis, that cell wall damage acts as a signal has been supported by experiments involving various cell wall-related mutants (Seifert and Blaukopf, 2010). Interestingly, 1- aminocyclopropane-1-carboxylic acid (ACC), a direct precursor in ethylene synthesis, responds to cell wall damage (De Cnodder et al., 2005; Tsang et al., 2011). Thus, we suggest that the signal derived from cell wall damage is weak in RNAi-10 and RNAi-16 fruit where softening is delayed, however, due to the higher level of cell wall integrity in the RNAi fruit. This would result in a lower level of ACC content relative to control fruit. *PpACO1* expression in RNAi-10 and RNAi-16 fruit was also inhibited (**Figure 6F**). Therefore, ethylene production was lower in these fruit, relative to the control fruit. Although all three hypotheses can explain the reduction in ethylene production observed in the RNAi-10 and RNAi-16 fruit, some unresolved issues remain, such as direct proof of the involvement of changes in galactose, OGA, and ACC contents in RNAi fruit and the identification of specific receptors of galactose and OGAs in cytomembranes. Confirmation of these hypotheses will thus require further complex experiments.

A reduction in ethylene production may delay peach fruit softening when the expression of *PpBGAL10* and *PpBGAL16* is down-regulated. The reduction in ethylene production, however, hardly affected β-gal activity in RNAi-10 and RNAi-16 fruit. These observations may suggest the existence of an indirect method of regulation between ethylene and *PpBGALs.* Ethylene can also regulate anthocyanin synthesis (El Kereamy et al., 2003; Cheng et al., 2016). Consequently, the inhibition of ethylene production may prevent anthocyanin synthesis and explain the lack of color change in the RNAi fruit where *PpBGAL10* and *PpBGAL16* are down-regulated (**Figure 3**).

## Conclusion

Our study demonstrated that *PpBGAL10* and -*16* are the main β-gal genes contributing to ethylene-dependent peach fruit softening. VIGS-induced down-regulation of *PpBGAL10* and *PpBGAL16* expression delays peach fruit softening by reducing PG and PME activity, which inhibits cell wall degradation and reduces ethylene production. The present study has provided strong evidence that β-gals play an important role in peach fruit softening.

## Author Contributions

MH and AW designed the experiments. HL, GL, and CS performed all plant physiological and molecular experiments. MQ and JL analyzed the data. HL and MQ wrote the manuscript. HL and CZ revised the manuscript.

## Conflict of Interest Statement

The authors declare that the research was conducted in the absence of any commercial or financial relationships that could be construed as a potential conflict of interest.
