# Supplementary material for: Down-Regulation of PpBGAL10 and PpBGAL16 Delays Fruit Softening in Peach by Reducing Polygalacturonase and Pectin Methylesterase Activity
Source: Front Plant Sci. 2018 Jul 11;9:1015. doi: 10.3389/fpls.2018.01015 (PMC6050397; doi:10.3389/fpls.2018.01015)
Supplement: Supplementary file 1 [file Table_1.DOCX]

| Gene name | Primers for qRT-PCR (5’→3’) | Gene name | Primers for qRT-PCR (5’→3’) |
| --- | --- | --- | --- |
| *PpBGAL11* | F: CATTAAGCTGGTTCAGGAGCACGG | *PpBGAL6* | F: CGCACTTCAGGAGGACCATTCA |
|  | R: GCAGTCCAGTTCTCAGTCCACAGA |  | R: GCACACGCTCCAGAAGAGTCAG |
| *PpBGAL13* | F: TAAGGCTATCCAGGAAGTCTATGC | *PpBGAL8* | F: GACAGTGCAAGAGCACTAGA |
|  | R: TCCGTTGTGAGGTTATTGTTGGT |  | R: TGGGTCACCAAATGTACTAAC |
| *PpBGAL9* | F: CCAGTGTGGTTGCGTGACATCC | *PpBGAL16* | F: GGGACTTGTGGGAGCTTTAG |
|  | R: AGCACCGAGACCTAGAGCCATC |  | R: ACGCTACAAGTCGTTGATCC |
| *PpBGAL4* | F: GAGCTGCTGGTCATGCCTACA | *PpBGAL3* | F: TGGGCGGCAAATATGGCTGTT |
|  | R: CTCCAAGCCTCGGTCCACAT |  | R: TCCTGAACTGGTCGTTGGTGAA |
| *PpBGAL17* | F: TGTCTTCGCTCTTCTACCTTCT | *PpBGAL15* | F: GCTATTTGGCTAAGGAAGATGGTTG |
|  | R:TTGCTCTCAACAGCCTATCTTC |  | R: GAGTGTTCTCACCGTCTTGCATAAA |
| *PpBGAL2* | F: TTCAGTCTCAAGGCGGTCCAAT | *PpBGAL12* | F: ACGCTCCTGTCATTGCTCTTGT |
|  | R: CCATCCAGTCCAGGCTTCAGTC |  | R: GCCACATCTCAGGTGTGCTTCT |
| *PpBGAL1* | F: TACAACACTGCGAGGCTTGGT | *PpBGAL5* | F: CAGGATGCTCCAGACCCAGTGA |
|  | R: CGATGGTGAGAAGAGGTGACTGT |  | R: GCCAGTCCAAGCCTCAGTCCAT |
| *PpBGAL7* | F: AAACAACATACCTGGTCAAGCGAA | *PpBGAL14* | F: GCTGGGAAAGGAAGGGCTCTCA |
|  | R: CGAACCACGGTCCATAGTTCTG |  | R: ATGGTGTCTCTGTTGGCGGTCA |
| *PpBGAL10* | F: GGCAATGGCGACGGCAAGTAA | *PpPG21* | F: TCCCTAATCAGCCAAATGTTCCAC |
|  | R: CATTAAGCTGGTTCAGGAGCACGG |  | R: GCCATCGGTGTTAGGGCTGTTC |
| *PpPME3* | F: CCAGACAATCATCACAGGAAACA | *PpACS2* | F: TGTTCAGCTCCCCGACTTTCAC |
|  | R: GCGTGTATAAGGTGTCTTGGTAGC |  | R: TCTTGCGGCCGATGTTCACC |
| *PpACO1* | F: CCCCCATGCGCCACTCCA |  |  |
|  | R: CATCACTGCCAGGGTTGTAAAAG |  |  |
| Gene name | Primers for VIGS cloning (5’→3’) | | |
| *PpBGAL16* | *F: TACCGAATTCTCTAGA*TCTGGCTCCATTCACTACCCTC | | |
|  | *R: ATGGAGGCCTTCTAGA*ATTAATATACTTCTGGGCCGCAGG | | |
| *PpBGAL10* | *F: TACCGAATTCTCTAGA*GGACTTTACAAGGGAGACGGTTTA | | |
|  | *R:ATGGAGGCCTTCTAGA*CCAAGATTCAAGGTCAATAGAGGG | | |

Supplementary Table 1. Primers sequence for PCR analysis.
